# Temporin-GHaK Exhibits Antineoplastic Activity against Human Lung Adenocarcinoma by Inhibiting the Wnt Signaling Pathway through miRNA-4516

**DOI:** 10.3390/molecules29122797

**Published:** 2024-06-12

**Authors:** Yueli Liu, Hui Liu, Jiaxin Zhang, Yingxia Zhang

**Affiliations:** 1 Key Laboratory of Tropical Biological Resources of Ministry of Education, School of Pharmaceutical Sciences, Collaborative Innovation Center of One Health, Hainan University, Haikou 570228, China; lyl20041981@126.com; 2 Key Laboratory of Tropical Translational Medicine of Ministry of Education & Key Laboratory of Brain Science Research Transformation in Tropical Environment of Hainan Province, School of Basic Medicine and Life Sciences, Hainan Medical University, Haikou 571199, China; huiliufaguo@163.com (H.L.); zhangjiaxin924098@163.com (J.Z.); 3 School of Life and Health Sciences, Hainan University, Haikou 570228, China

**Keywords:** GHaK, lung adenocarcinoma, miR-4516, Wnt signaling pathway

## Abstract

(1) Background: GHaK is derived from the antimicrobial peptide temporin-GHa by substituting the amino acid H with K to enhance its bactericidal activity. The present research aims to broaden the pharmacological potential of GHaK by exploring its antineoplastic activity against human lung adenocarcinoma. (2) Methods: The cell viability, migration, invasion, apoptosis, and cell cycle of A549 and PC-9 cells were tested after GHaK treatment. miRNA sequencing, RT-PCR, Western blotting, and luciferase reporter gene assay were further performed to reveal the potential mechanism. (3) Results: GHaK significantly suppressed cell viability, migration, and invasion; induced apoptosis; and caused cell cycle arrest in the G2/M and S phase in PC-9 and A549 cells, respectively. The miRNA sequencing results show a total of 161 up-regulated and 115 down-regulated miRNAs. Furthermore, the study identified six up-regulated miRNAs (miR-4516, miR-4284, miR-204-5p, miR-12136, miR-4463, and miR-1296-3p) and their inhibitory effects on the expressions of target genes (Wnt 8B, FZD2, DVL3, and FOSL1) caused by miR-4516 directly interacting with Wnt 8B. Western blotting revealed the down-regulation of p-GSK-3β, along with a decreased expressions of cyclin A1 and CDK2 in A549 cells and cyclin B1 and CDK1 in PC-9 cells. (4) Conclusions: Temporin-GHaK exhibits antineoplastic activity against human lung adenocarcinoma by inhibiting the Wnt signaling pathway through miRNA-4516.

## 1. Introduction

Non-small cell lung cancer (NSCLC) is one of the leading causes of mortality in most countries. Although great achievements have been made in the diagnosis and therapy of NSCLC recently, the overall 5-year survival rate remains less than 5% [1]. NSCLC consists of three histologic subtypes, namely lung adenocarcinoma (LUAD), squamous cell carcinoma, and large cell carcinoma, with LUAD being the predominant subtype [2]. NSCLC is frequently diagnosed at an advanced stage, resulting in limited access to effective and curative therapies for patients [3]. Small-molecule tyrosine kinase inhibitors, working together with immunotherapy, can improve the prognosis of NSCLC, especially in cases of metastatic disease. However, multi-drug resistance abolishes their benefit; therefore, there is an emergent need for new medicines to improve NSCLC therapy [4,5]. Yu HH et al. investigated the use of the marine antimicrobial peptide (AMP) epinecidin-1 as a potential treatment for NSCLC and demonstrated its effectiveness in inhibiting tumor cell growth [6].

AMPs are naturally occurring small peptides with less than 50 amino acids, often found in bacteria, archaea, protists, fungi, plants, and animals. They can eradicate invading pathogens and therefore play a critical role in the innate immune system [6,7,8,9]. Many AMPs have demonstrated antineoplastic properties. For instance, moricin exhibited inhibitory effects on human triple-negative breast cancer growth [10]. Br-J-I and LL37 were found to prevent the progression of colorectal cancer [11,12]. SKACP003 contributed to decreasing the advancement and prognosis of breast cancer [13]. ATMP5 and RT2 effectively arrested the growth of the MDA-MB-231 and Caco-2 cells, respectively [14,15]. Temporin-GHa (GHa) is a kind of AMP extracted from the skin of *Hylarana guentheri*, which is positively charged with histidine and exhibits antibacterial activity against *Staphylococcus aureus* [16]. To improve its antibacterial effect, we synthesized a GHa analog—GHaK—by substituting the amino acid H with K. Our findings demonstrate that GHaK exhibited increased antibacterial activity against Gram-positive bacteria such as *Staphylococcus aureus* and *Bacillus subtilis*, as well as Gram-negative bacteria such as *Escherichia coli* and *Pseudomonas aeruginosa* [17,18]. Brevinin-1RL1, a naturally occurring AMP derived from frog skin secretions, triggered apoptosis in various tumor cell lines such as MDA-MB-231, HCT116, A549, SW480, and SMMC-7721 [19]. Given that GHaK is a derivative of GHa, a peptide also extracted from frog skin secretions, and considering the known anti-cancer effects of the epinecidin-1 against LUAD, we are keen to investigate the potential antineoplastic activity of GHaK against this type of cancer. In this study, human LUAD cells, namely A549 and PC-9, were used to explore its antineoplastic activity and the underlying mechanisms.

## 2. Results

### 2.1. GHaK Suppressed the Cell Viability of PC-9 and A549 Cells without Obvious Inhibition of HaCaT Cells

**Figure 1:**
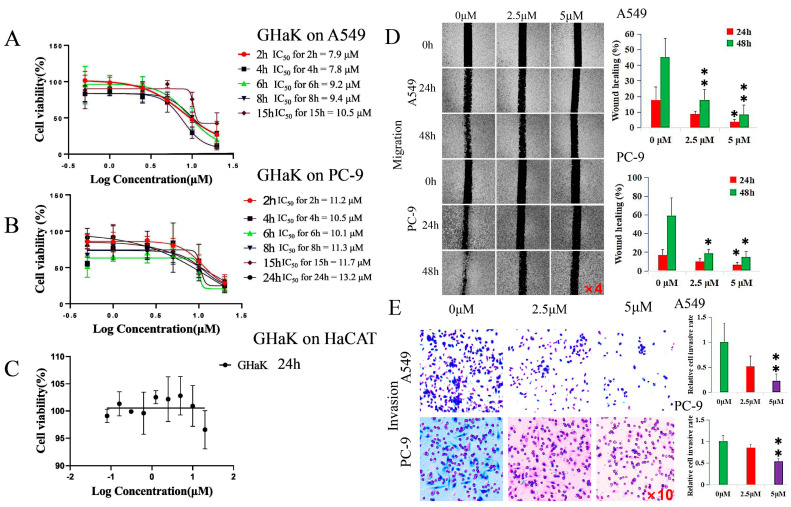
GHaK suppressed the cell viability, migration, and invasion of PC-9 and A549 cells. (**A**,**B**). GHaK suppressed the cell viability of A549 and PC-9 cells at 2, 4, 6, 8, and 15 h with an additional inhibition on PC-9 cells at 24 h with the lowest IC_50_ value of 7.8 μM at 4 h for A549 and of 10.1 μM at 6 h for PC-9 cells. (**C**) GHaK did not reduce the cell viability of HaCaT cells after 24 h of treatment. (**D**) GHaK inhibited the migration of PC-9 and A549 cells at the dose of 5 μM at 48 h (for PC-9 cells, *t* = 3.86, *p* < 0.05; for A549 cells, *F* = 14.77, *p* < 0.01) and at 24 h (for PC-9 and A549 cells, *F* = 5.17, 5.74, respectively, *p* < 0.05) with an additional inhibition at the dose of 2.5 μM at 48 h (for PC-9 cells, *t* = 3.59, *p* < 0.05; for A549 cells, *F* = 14.77, *p* < 0.01). The pictures were magnified by 4 times. (**E**) GHaK inhibited the invasion of PC-9 (*t* = 12.75, *p* < 0.01) and A549 cells (*t* = 10.11, *p* < 0.01) at the dose of 5 μM after 24 h of treatment. (* *p* < 0.05; ** *p* < 0.01). The pictures were magnified by 10 times.

CCK-8 assay was employed to determine the effects of GHaK on the cell viability of A549 and PC-9 cells and its cytotoxicity on HaCaT cells in vitro. The results show that GHaK exhibited a reduced cell viability of A549 and PC-9 cells at 2, 4, 6, 8, and 15 h and an additional inhibition on PC-9 cells at 24 h in a dose-dependent manner. GHaK achieved its biggest potency with an IC_50_ value of 7.8 μM at 4 h for A549 and with an IC_50_ value of 10.1 μM at 6 h for PC-9 cells. It did not reduce the cell viability of HaCaT cells significantly (Figure 1A–C).

### 2.2. GHaK Inhibited the Migration and Invasion of PC-9 and A549 Cells

Wound-healing and transwell assays were used to demonstrate whether GHaK exerted suppressive effects on the migration and invasion of A549 and PC-9 cells. GHaK at the dose of 5 μM significantly inhibited the wound-healing activity of PC-9 and A549 cells at 48 h (for PC-9 cells, *t* = 3.86, *p* < 0.05; for A549 cells, *F* = 14.77, *p* < 0.01) and at 24 h (for PC-9 and A549 cells, *F* = 5.17, 5.74, respectively, *p* < 0.05) with an additional inhibition at the dose of 2.5 μM at 48 h (for PC-9 cells, *t* = 3.59, *p* < 0.05; for A549 cells, *F* = 14.77, *p* < 0.01) in a concentration- and time-dependent manner (Figure 1D). GHaK greatly suppressed the invasion of PC-9 cells (*t* = 12.76, *p* < 0.01) and A549 cells (*t* = 10.11, *p* < 0.01) at the dose of 5 μM after 24 h of treatment (Figure 1E).

### 2.3. GHaK Induced Apoptosis and Arrested Cell Cycle in PC-9 and A549 Cells

**Figure 2:**
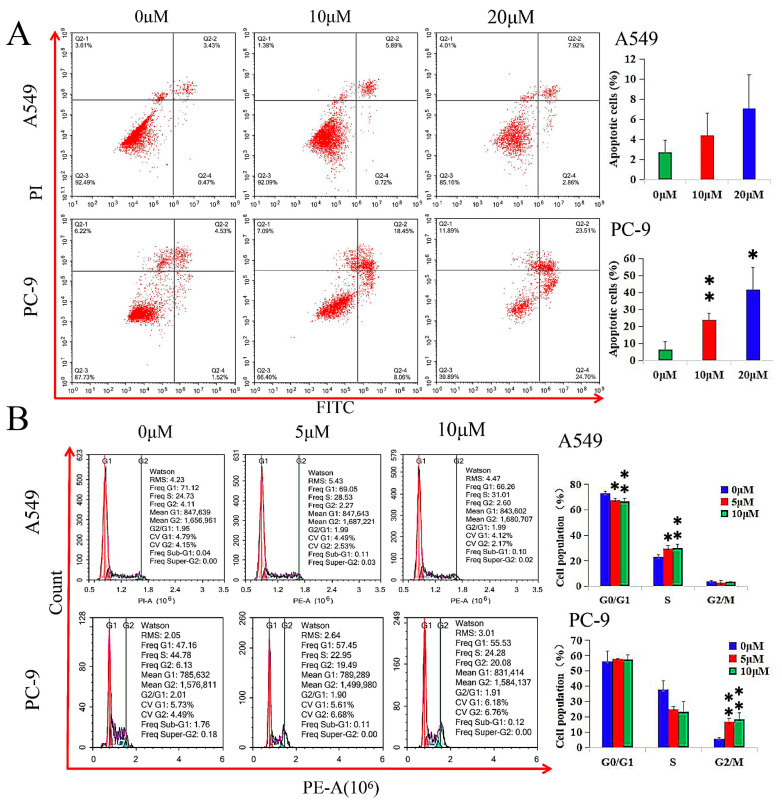
GHaK induced apoptosis and arrested cell cycle in PC-9 and A549 cells. (**A**) GHaK induced apoptosis in PC-9 cells (at the dose of 10 μM, *t* = −5.21, *p* < 0.01; at the dose of 20 μM, *t* = −4.39, *p* < 0.05) after 20 h of treatment without significant induction of apoptosis in A549 cells at both doses of 10 and 20 μM (*F* = 2.55, *p* > 0.05) after 15 h of treatment. (**B**) GHaK led to a lower G0/G1 population percentage (*F* = 9.00, *p* < 0.05) in A549 cells and arrested the cell cycle in the S phase (*F* = 8.74, *p* < 0.05) and in the G2/M phase (*F* = 20.86, *p* < 0.01) at the doses of 5 and 10 μM after 15 and 20 h of treatment in A549 and PC-9 cells, respectively. (* *p* < 0.05; ** *p* < 0.01).

GHaK induced apoptosis in PC-9 cells (at the dose of 10 μM, *t* = −5.21, *p* < 0.01; at the dose of 20 μM, *t* = −4.39, *p* < 0.05) after 20 h of treatment, while it did not exhibit significant induction in apoptosis in A549 cells in both doses of 10 and 20 μM (*F* = 2.55, *p* > 0.05) after 15 h of treatment (Figure 2A).

GHaK led to a lower G0/G1 population percentage (*F* = 9.00, *p* < 0.05) in A549 cells and arrested the cell cycle in the S phase (*F* = 8.74, *p* < 0.05) and in the G2/M phase (*F* = 20.86, *p* < 0.01) at the doses of 5 and 10 μM after 15 and 20 h of treatment in A549 and PC-9 cells, respectively (Figure 2B).

### 2.4. GHaK Caused Differential Expressions of miRNA with GO and KEGG Analyses

**Figure 3:**
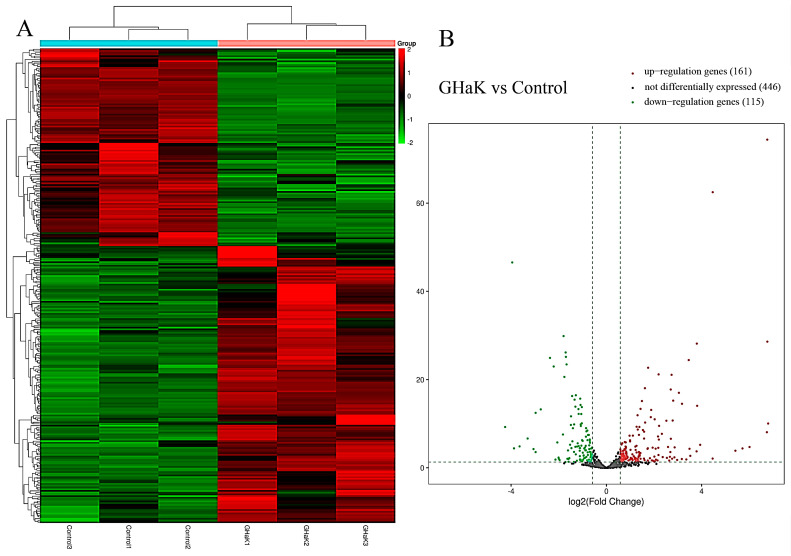
Heatmap and volcano plot showing differential expressions of miRNAs after GHaK treatment. (**A**) Heatmap showing differential expressions of miRNAs caused by GHaK treatment for 20 h on PC-9 cells. (**B**) Volcano plots exhibiting a total of 446 miRNAs that were identically expressed, 161 miRNAs that were up-regulated, and 115 miRNAs that were down-regulated after GHaK treatment for 20 h on PC-9 (log2 (fold change) > 1.5, *p* < 0.05).

**Figure 4:**
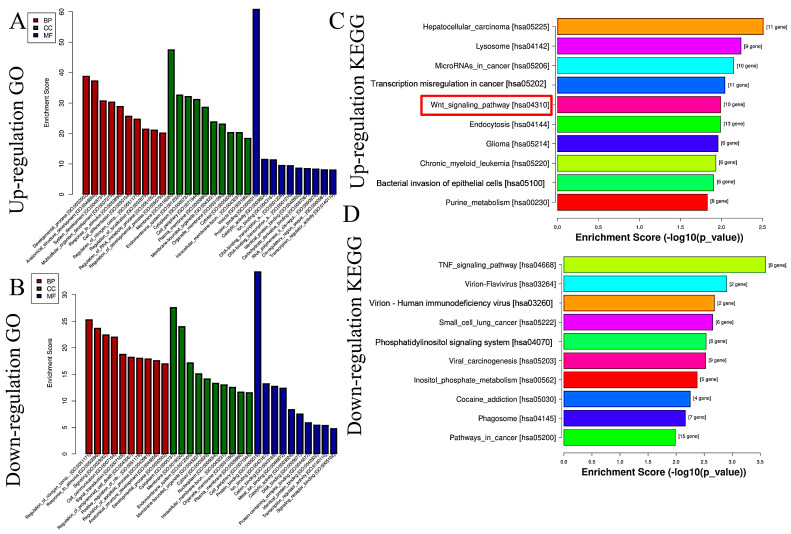
GO and KEGG analyses of differential expressions of miRNAs caused by GHaK treatment. (**A**). GO analysis exhibited up-regulated miRNAs caused by GHaK treatment on PC-9 cells in terms of the top 10 categories in BB, CC, and MF. (**B**). GO analysis exhibited down-regulated miRNAs caused by GHaK treatment on PC-9 cells in terms of the top 10 categories in BB, CC, and MF. (**C**). KEGG analysis showed up-regulated miRNAs distributed in the top 10 pathways caused by GHaK treatment on PC-9 cells. (**D**). KEGG analysis showed down-regulated miRNAs distributed in the top 10 pathways caused by GHaK treatment on PC-9 cells. Red box indicated the pathway would be validated.

Based on miRNA sequencing data (miRNA-seq), a total of 722 miRNAs were selected for paired comparisons, as shown in Figure 3A. Among these, 161 miRNAs were up-regulated and 115 miRNAs were down-regulated (Figure 3B). The most enriched categories in terms of up- or down-regulated biological processes (BPs) were the development process, anatomical structure development, and system development and the nitrogen compound metabolic process, response to stimulus, and response to signaling, respectively. In terms of up- or down-regulated cellular components (CCs), the most enriched categories were the membranes, endomembrane system, and cytoplasm and the cytoplasm, membrane, and endomembrane system, respectively. In terms of the up- or down-regulated molecular functions (MFs), the most enriched categories were protein binding, catalytic activity, ion binding, and protein binding and ion binding and cation binding, respectively (Figure 4A,B). KEGG enrichment analysis showed that differentially up-regulated miRNAs were widely distributed in the top five pathways of hepatocellular carcinoma, lysosome, microRNAs in cancer, transcription misregulation in cancer, and Wnt signaling pathway, and the differentially down-regulated miRNAs were extensively distributed in the top five pathways of the TNF signaling pathway, vivion flavivirus, virion–human immunodeficiency, small cell lung cancer, and phosphatidylinositol signaling system (Figure 4C,D).

### 2.5. Validation of Differentially Expressed miRNAs and Their Target Genes Caused by GHaK Treatment

**Figure 5:**
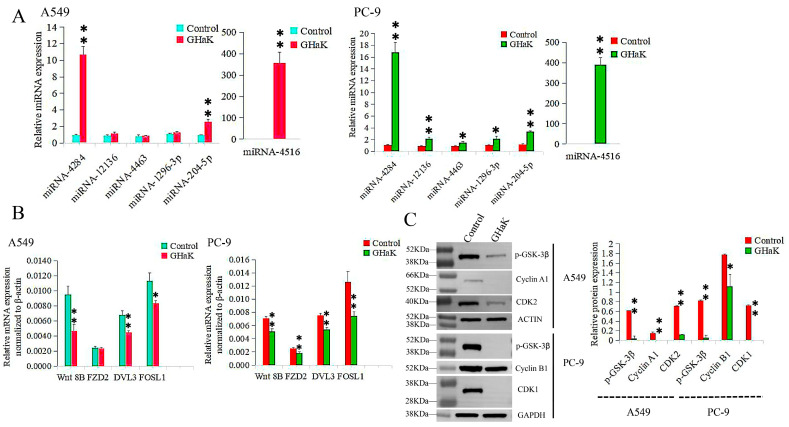
RT-PCR and Western blotting validated differentially expressed miRNAs involved in the Wnt signaling pathway caused by GHaK treatment and expressions of their target genes. (**A**). RT-PCR analysis confirmed up-regulations of miR-4516, miR-4284, and miR-204-5p in PC-9 (*t* = −18.74, −16.26, and −18.07, respectively, *p* < 0.01) and A549 cells (*t* = −12.00, −17.64, and −9.70, respectively, *p* < 0.01) with additional up-regulations of miR-12136, miR-4463, and miR-1296-3p in PC-9 cells (*t* = −8.70, *p* < 0.01; *t* = −4.32, −4.24, *p* < 0.05) caused by GHaK treatment. (**B**). RT-PCR analysis confirmed down-regulations of the target genes Wnt 8B, DVL3, and FOSL1 in both PC-9 (*t* = 6.83, 7.98, and 5.30, respectively, *p* < 0.01) and A549 cells (*t* = 5.68, 6.49, *p* < 0.01; *t* = 4.49, *p* < 0.05) with an additional down-regulation of FZD2 in PC-9 cells (*t* = 5.00, *p* < 0.01) involved in the Wnt signaling pathway caused by GHaK treatment. (**C**). Western blotting analysis confirmed down-regulation expressions of p-GSK-3β, cyclinA1, and CDK2 in A549 cells (*t* = 19.46, 46.00, and 44.50, respectively, *p* < 0.01) and p-GSK-3β, cyclinB1, and CDK1 in PC-9 cells (*t* = 25.36, *p* < 0.01; *t* = 4.52, *p* < 0.05; *t* = 72.00, *p* < 0.01) involved in the Wnt signaling pathway caused by GHaK treatment. (* *p* < 0.05; ** *p* < 0.01).

Differentially expressed miRNAs that targeted the Wnt signaling pathway were sequenced and validated by RT-PCR. miR-4516, miR-4284, and miR-204-5p were confirmed to be up-regulated in PC-9 (*t* = −18.74, −16.26, and −18.07, respectively, *p* < 0.01) and A549 cells (*t* = −12.00, −17.64, and −9.70, respectively, *p* < 0.01) with additional up-regulations of miR-12136, miR-4463, and miR-1296-3p in PC-9 cells (*t* = −8.70, *p* < 0.01; *t* = −4.32, −4.24, *p* < 0.05) (Figure 5A). Their target genes, Wnt 8B, DVL3, and FOSL1, were down-regulated in both PC-9 (*t* = 6.83, 7.98, and 5.30, respectively, *p* < 0.01) and A549 cells (*t* = 5.68, 6.49, *p* < 0.01; *t* = 4.49, *p* < 0.05), with an additional down-regulation of FZD2 in PC-9 cells (*t* = 5.00, *p* < 0.01) (Figure 5B).

### 2.6. GHaK Depressed the Wnt Signaling Pathway by Reducing the Levels of Some Key Proteins

To investigate the inhibitory effects of GHaK on some key proteins in the Wnt signaling pathway, Western blot analysis was conducted to examine the expressions of specific proteins in PC-9 and A549 cells. p-GSK-3β, cyclinA1, and CDK2 were down-regulated in A549 cells (*t* = 19.46, 46.00, and 44.50, respectively, *p* < 0.01) with reduced expressions of p-GSK-3β, cyclinB1, and CDK1 in PC-9 cells (*t* = 25.36, *p* < 0.01; *t* = 4.52, *p* < 0.05; *t* = 72.00, *p* < 0.01) following treatment with GHaK (Figure 5C). GHaK may impact the Wnt signaling pathway by influencing the expressions of key proteins involved in the cell cycle and regulation of apoptosis.

### 2.7. miR-4516 Directly Combined with Wnt 8B

The prediction from TargetScan 8.0 and miRDB that the Wnt 8B gene contained four miR-4516 binding sites in the 3′-UTR region aligned with the experimental findings from the luciferase reporter assay. The assay demonstrated that when miR-4516 mimics were co-transfected with the wild-type (WT) Wnt 8B construct, there was a significant decrease in the ratio of firefly luciferase to renilla luciferase compared to the control group (*t* = 9.94, *p* < 0.001). This indicated that the binding of miR-4516 to the 3′-UTR of the Wnt 8B gene led to an inhibition of luciferase expression, supporting the notion that miR-4516 directly targeted Wnt 8B.

**Figure 6:**
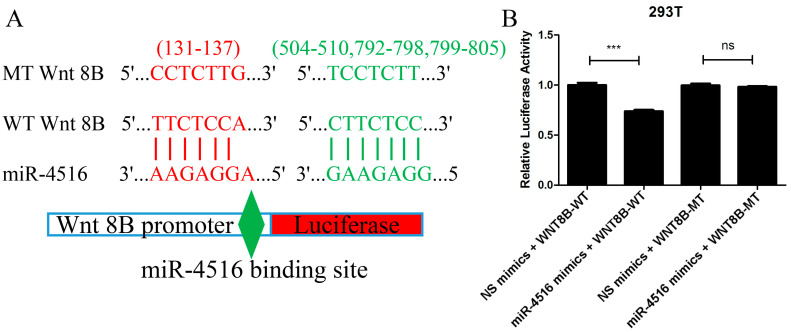
Luciferase reporter assay confirming the combination of miR-4516 with Wnt 8B. (**A**). A total of four miRNA-4516 binding sites (131-137, 504-510, 792-798, and 799-805) in the 3′-UTR of the Wnt 8B gene were mutated. Wnt 8B promoter, either wild-type or mutant-type Wnt 8B, in the miR-4516 binding sites were cloned into pmirGLO reporter vectors upstream of the luciferase reporter gene. (**B**). miR-4516 mimics and NC mimics were synthesized and mixed with cloned pmirGLO reporter vectors in 50μL of basic DMEM. 293T cells were cultured and co-transfected with NC mimics + Wnt 8B-WT, miR-4516 mimics + Wnt 8B-WT, NC mimics + Wnt 8B-MT, and miR-4516 mimics + WNT 8B-MT using LipofectamineTM 2000 (Invitrogen, Carlsbad, CA, USA) for 4 h. miR-4516 mimics + Wnt 8B-WT construct showed a significant decrease (*t* = 9.94, *p* < 0.001), and miR-4516 mimics + Wnt8B MT construct showed a lack of difference in the ratio of firefly luciferase to renilla luciferase compared to the control group. (*** *p* < 0.001). “ns” standed for “no statistically significant difference”.

Furthermore, the lack of difference in the luciferase ratio between the miR-4516 mimics + mutant-type (MT) Wnt8B construct and the control suggested that the binding site mutation in the Wnt 8B gene disrupted the combination between miR-4516 and its target site, abolishing the inhibitory effect observed with the WT Wnt 8B construct (Figure 6).

### 2.8. Expressions of miR-4516 and Its Target Genes in the Wnt Signaling Pathway in Human Lung Adenocarcinoma

**Figure 7:**
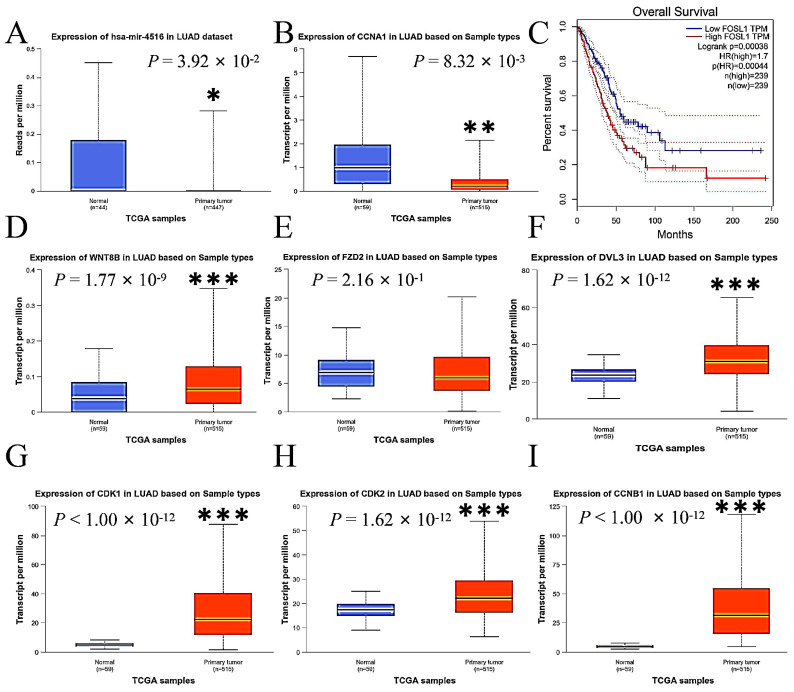
The comparisons of expression levels of miR-4516, Wnt 8B, FZD2, DVL3, FOSL1, and other key genes associated with cell cycle and apoptosis in primary LUAD tumor tissue with those in normal lung tissue using data from the TCGA and GTEx databases in the human samples. (**A**). miR-4516 was down-regulated in the primary LUAD tumor tissue compared to normal lung tissue in the human samples (*p* < 0.05). (**B**,**D**–**I**). The expressions of CCNA1, Wnt 8B, DVL3, CDK1, CDK2, and CCNB1 were up-regulated in the primary LUAD tumor tissue compared to normal lung tissue in the human samples (*p* < 0.01; *p* < 0.001). (**C**). Overall survival was inversely associated with FOSL1 expressions in patients with LUAD (*p* < 0.001). (* *p* < 0.01; ** *p* < 0.01; *** *p* < 0.001).

This study compared the expression levels of miR-4516, Wnt 8B, FZD2, DVL3, FOSL1, CDK1, CDK2, CCNA1, and CCNB1 in primary LUAD tumor tissue with those in normal lung tissue using data from The Cancer Genome Atlas (TCGA) database to assess the consistency of our findings with the human sample. The expressions of miR-4516 were lower (Figure 7A), while the expressions of Wnt 8B, DVL3, FOSL1, CDK1, CDK2, CCNA1, and CCNB1 were enhanced in tumor tissue compared to normal lung tissue in the human samples (Figure 7B,D–I). Additionally, overall survival was inversely associated with expressions of FOSL1 in patients with LUAD according to data from the TCGA and the Genotype-Tissue Expression (GTEx) databases by Gene Expression Profiling Interactive Analysis (http://gepia.cancer-pku.cn/, accessed on 5 March 2024) (Figure 7C). 

## 3. Discussion

Multi-drug resistance is a significant challenge in the treatment of NSCLC, prompting researchers to seek alternative treatment options. AMPs have gained attention from researchers due to their anti-cancer properties. GHa consists of 13 amino acid residues derived from the skin of frogs inhabiting Hainan Island, China. To generate GHaK, modifications were made by replacing histidine residues with lysine residues at both ends of the GHa peptide. Specifically, the modifications led to a significant increase in antimicrobial activity against *Staphylococcus aureus*. The enhanced positive charges on the hydrophilic surface may have improved the peptide’s ability to interact with and disrupt the cell membranes of the bacteria, leading to increased antimicrobial efficacy [17,18]. As mentioned above, many AMPs target ROS-mediated caspase-dependent apoptosis, Wnt signaling pathway, and mitochondrial voltage-dependent anion channels to exert their anti-cancer activity [10,13,20]. However, the functions of GHaK against LUAD and its underlying mechanisms remain unclear.

In this study, A549 and PC-9 cells were used to examine the antineoplastic activity of GHaK. GHaK significantly decreased cell viability, reduced migration and invasion, induced apoptosis, and arrested cell cycle in a concentration-dependent manner on PC-9 and A549 cells without obvious cytotoxicity on HaCaT cells, indicating that GHaK might be an ideal candidate for the treatment of NSCLC.

To further study its mechanisms, miRNA-seq was employed and the findings show that GHaK altered miRNA expressions, particularly impacting the miRNAs involved in the Wnt signaling pathway; miR-4516 was one of the most up-regulated. Numerous studies have demonstrated the involvement of miR-4516 in many kinds of cancer progression. For example, decreased levels of miR-4516 have been linked to enhanced FOSL1-mediated proliferation and aggressiveness in triple-negative breast cancer [21]. Additionally, the interaction of miR-4516 and lncRNA PART1 led to a reduced miR-4516 expression and the promotion of breast cancer development [22]. The over-expression of miR-4516 prevented pancreatic cancer progression by negatively regulating orthodenticle homeobox 1 [23]. Moreover, the miR-4516/SOX5 axis was involved in cell proliferation and invasion in human hepatocellular carcinoma [24]. Over all, decreased levels of miR-4516 were observed to promote tumor cell proliferation, migration, and invasion, ultimately facilitating the advancement of cancer. Our research reveals that GHaK substantially increased the expression of miR-4516, providing insight into how GHaK functions in LUAD cells.

Prior research has established a connection between miR-4516 and FOSL1-driven tumor cell proliferation and aggressiveness in breast cancer. It was discovered that Wnt 8B acts as an upstream regulator of FOSL1 and has been down-regulated after combination with miR-4516 in LUAD cells. In detail, the significant enhancement of miR-4516 by GHaK and its ability to bind to the 3′-UTR of the Wnt 8B gene led to the inhibition of Wnt 8B expression. The downstream effects of GHaK, particularly the down-regulation of downstream genes of Wnt 8B, such as FZD2, DVL3, and FOSL1, provided important insights into the molecular mechanisms underlying GHaK’s impacts on cell proliferation, migration, and invasion in LUAD cells.

The Wnt signaling pathway plays an important role in the development of LUAD [25,26,27,28,29,30,31]. The phosphorylation of GSK-3β was a critical step in the activation of the Wnt signaling pathway [32,33]. The down-regulation of p-GSK-3β caused by GHaK in both A549 and PC-9 cells resulted in the reduced expressions of cyclin A1, CDK2, and cyclin B1, CDK1, supporting GHaK’s effects on cell cycle arrest and the induction of apoptosis. 

Cyclin A1, CDK2, and cyclin B1, CDK1, were responsible for the progression of the cell cycle, especially in the S and G2/M phases, respectively. GHaK reduced expressions of cyclin A1, CDK2, in A549 cells and cyclin B1, CDK1, in PC-9 cells and led to cell cycle arrest in different phases. This phenomenon is probably related to different intracellular metabolisms according to the different IC_50_ values and duration of action in A549 and PC-9 cells. More research needs to be conducted to verify this mechanism.

According to human data derived from the TCGA and GTEx databases, decreased expressions of miR-4516 and increased expressions of Wnt 8B, DVL3, FOSL1, CCNA1, CCNB1, CDK1, and CDK2 in tumor tissue contributed to the progression of LUAD. In addition, FOSL1 was reported to be a prognostic marker and potential therapeutic target inversely associated with overall survival of patients with LUAD [34,35,36,37]. These altered expressions greatly support GHaK’s potential antineoplastic activity against LUAD. Although data from various databases support the anti-cancer properties of GHaK, various complex potential confounding factors such as age, gender, smoking and drinking habits, gene mutations, or coexisting diseases may influence the expressions of miR-4516, Wnt 8B, DVL3, FOSL1, CCNA1, CCNB1, CDK1, and CDK2 in tumor tissue. Validation studies are essential to enhance the reliability of these findings.

Our research confirms the effectiveness of GHaK in combating LUAD, suggesting its promise as a valuable treatment option for NSCLC. However, there are certain research gaps that need to be addressed. These include a lack of comprehensive understanding of GHaK’s metabolism and its active form. Furthermore, there is a need for additional data on the impact of GHaK on various NSCLC cell lines, such as H1299, H460, and H650. In addition, future studies will need to include in vivo experiments to validate the antineoplastic efficacy of GHaK in inhibiting tumor cell growth in live animal models. These areas will be the focus of upcoming research efforts to further establish the clinical potential of GHaK.

Based on our previous results, GHaK demonstrated 10% hemolysis of human red blood cells (hRBCs) at a dose of 16 μM, reaching 50% hemolysis at 66 μM [17]. This signifies that the dose of 10 μM of GHaK, known for its antineoplastic properties, exhibits minimal toxicity toward hRBCs, making it a promising candidate for further in vivo evaluation. With its solubility in water and a molecular weight of 1446.83, GHaK has the ability to reach the lungs via the bloodstream in live animals and it easily penetrates cell membranes, enhancing its potential as an antineoplastic candidate. Intravenous injection or nasal inhalation can be employed to deliver GHaK, and appropriate dosage regimens should be acquired through in vivo experiments to achieve its effective concentration in lungs.

In conclusion, GHaK intensively increased expressions of miRNA-4516 targeting the Wnt signaling pathway and successively inhibited its activation. Consequently, this has led to GHaK’s antineoplastic activity against LUAD by decreasing cell viability, reducing migration and invasion, inducing apoptosis, and arresting the cell cycle. Temporin-GHaK exhibits antineoplastic activity against human LUAD by inhibiting the Wnt pathway through miRNA-4516.

## 4. Materials and Methods

### 4.1. Cell Culture

A549 and PC-9 cell lines derived from human LUAD were purchased from the Cell Bank of the Chinese Academy of Sciences (Shanghai, China) and cultured in high-glucose DMEM (HG DMEM) medium (Viva Cell, Shanghai, China, REF: C3113-0500) with 10% fetal bovine serum (NEWZERUM, Christchurch, New Zealand, Cat No: FBS-S500). HaCaT cells were cultured in DMEM (Gibco, Beijing, China, REF: C11995500BT) to detect cytotoxicity of GHaK.

### 4.2. CCK-8 Assay

A549, PC-9, and HaCaT cells were inoculated in a 96-well plate with 5000 cells in each well. GHaK was dissolved in concentrations of 0.5, 1, 2.5, 5, 10, and 20 μM HG DMEM. Cells were treated with each dose for 2, 4, 6, 8, and 15 h for A549 and PC-9 cells, with an additional 24 h treatment for PC-9 cells. HaCaT cells were treated in doses of 0.08, 0.16, 0.31, 0.63, 1.25, 2.5, 5.0, 10, and 20 μM for 24 h. Thereafter, each cell was washed with PBS twice and filled with 100 ul of basal medium. Moreover, 10 μL of CCK-8 (Biosharp, Beijing, China, Cat No: BS350A) was added to each well and incubated for 30 min, 40 min, and 3 h for PC-9, A549, and HaCaT cells, respectively. The optical density was recorded at 450 nm using a microplate reader. All experiments were replicated three times, and the average value from the three replications was used to calculate cell viability (%).

### 4.3. Wound-Healing Assays

Prior to conducting the wound-healing assays, this study verified that treatment of GHaK at the dose of 5 μM for 24 h did not result in cytotoxic effects on PC-9 and A549 cells, shown in Figure S1 (Supplementary Materials). Then, 1 × 10^4^ cells of A549 or PC-9 were placed in an ibidi culture-insert. When cell confluence reached 100%, the ibidi culture-insert was removed to generate a wound. Subsequently, cells were treated with GHaK in doses of 2.5 and 5 μM for 24 h. To measure the wound, a picture of the wound was taken at 0, 24, and 48 h after treatment and measured using Image J software V1 8.0. The wound-healing percentage was calculated. All experiments were replicated three times.

### 4.4. Transwell Assay

The upper chamber was coated with 50 μL of 25% Matrigel (Solarbio, Beijing, China, Cat No: M8370) and then placed in a cell incubator for 30 min to conduct Matrigel-based transwell assays. A549 or PC-9 cells were diluted in basal HG DMEM medium to a concentration of 1.25 × 10^5^/mL; then, 100 μL of the cell suspension was added and treated with GHaK in doses of 2.5 and 5 μM in the upper chamber for 24 h. Moreover, 500 μL of HG DMEM medium with 10% FBS was added to the bottom chamber. Cells were permitted to invade through the Matrigel-coated 8.0 µm membrane pores. The invading cells were washed with PBS twice and then fixed in 4% paraformaldehyde for 20 min and stained successively with 0.1% crystal violet (Solarbio, Cat No: G1064) for another 20 min. The invading cells were photographed and counted under a microscope, and the cell counts were used for analysis.

### 4.5. Flow Cytometry Assay

The density of A549 or PC-9 cells was adjusted to 1.5 × 10^4^ per mL, and then samples were placed in a 6-well plate with 3 × 10^4^ cells per well. Cells were cultured for 24 h and then treated with GHaK in doses of 10 and 20 μM to test cell apoptosis or 5 and 10 μM to test the cell cycle in A549 cells for 15 h and PC-9 cells for 20 h, respectively. Cells were digested with 0.25% trypsin (Gibco, Canada, Cat No: 2509042) and then centrifuged with 1000 rpm for 5 min; the supernatant fluid was discarded. The cells were washed with cold PBS twice before analysis. All experiments were replicated three times.

Cell apoptosis assay: A commercial apoptosis detection kit (BD, San Diego, CA, USA, Cat No: 556547) was used to measure cell apoptosis. The cell suspension was prepared with a cell density of 1 × 10^6^ cells per mL in 1 × binding buffer. Cell suspension was added to 4 tubes with 100 μL in each. Three of the four tubes were incubated with 5 μL annexin V-FITC, 5 μL PI, or a combination of 5 μL annexin V-FITC with 5 μL PI for 15 min at 25 °C in the dark according to the instruction of the manufacturer. The sample was measured via flow cytometry within 1 h.

Cell cycle assay: A commercial cell cycle detection kit (BD, San Diego, CA, USA, Cat No: 550825) was used to detect cell cycle distribution. First, 75% alcohol was used to fix the cells at 4 °C overnight, and then the cells were centrifuged (1000 rpm, 5 min). After removing the supernatant, the cells were washed and then resuspended with 0.5 mL PBS and then incubated with 0.25 mL PI at 25 °C for 30 min in the dark. The sample was analyzed via flow cytometry within 1 h.

### 4.6. miRNA Sequencing

PC-9 cells were treated with GHaK at the dose of 10 μM for 20 h. Total RNA was extracted by using a Trizol reagent kit (Invitrogen, Carlsbad, CA, USA, REF:15596026CN). After quality control with 1% agarose gels and NanoDrop^®^ ND-1000, RNA libraries were constructed and the eligible libraries were sequenced using the Illumina platform to generate single-ended reads at Shanghai Kangsheng Biotech company. After quality control, raw sequencing data were removed using 3′adapters with cutadapt, and reads with lengths shorter than 17 nucleotides were discarded. Trimmed reads were mapped with the bowtie program. 

The mapped reads were further identified as mature and precursor miRNAs with mirdeep2. Clean reads with average counts per million (CPMs) > 1 were analyzed. EdgeR was employed to analyze the differential expressions of the clean reads. The criteria for differential expressions were a *p* value < 0.05 and a log2 (fold change) > 1.5.

Target genes of differentially expressed miRNAs were predicted using miRDB (https://mirdb.org/) and TargetScan (https://www.targetscan.org/, accessed on 1 October 2023). Subsequently, Gene Ontology (GO) enrichment (http://www.geneontology.org) and KEGG pathway analyses (https://www.kegg.jp/, accessed on 10 October 2023) were conducted using a *p* value threshold for FDR correction set to 0.05.

### 4.7. RT-PCR Assay

Cells were treated with GHaK at the dose of 10 μM in A549 cells for 15 h and 10 μM in PC-9 cells for 20 h. RT-PCR was employed to validate differentially expressed miRNAs and their target genes. Total RNA was extracted, and cDNA was constructed according to the abovementioned procedure. RT-PCR procedures were performed with QuantStudio™ 5 Real-time PCR System (Applied Biosystems, Waltham, MA, USA). The cycling conditions consisted of a denaturation program (95 °C, 10 min), an amplification and quantification program repeated 40 times (95 °C for 10 s, 60 °C for 60 s), a melting curve program (95 °C for 10 s, 60 °C for 60 s, and 95 °C for 15 s, 60–95 °C with a heating rate of 0.05 or 0.075 °C per second, and a continuous fluorescence measurement), and finally, a cooling step to 40 °C. 

Expression levels of predicted differentially expressed miRNAs, including miR-4516, miR-4284, miR-204-5p, miR-12136, miR-4463, and miR-1296-3p, were examined using U6 snRNA as a reference. Cycle values (Cts) were used to analyze miRNA expression levels. Fold-change measurements of the GHaK-treated groups were compared with the control samples using the 2^−ΔΔCt^ formula. The primer sequences are listed in Table S1 (Supplementary Materials).

Expression levels of predicted target genes, including Wnt 8B, FZD2, DVL3, and FOSL1, were measured with β-actin as a reference. The ratio of the quantity of the target genes to β-actin was used to analyze the expression levels of target genes. The primer sequences are listed in Supplementary Table S2 (Supplementary Materials).

### 4.8. Western Blotting Assay

After being treated with GHaK at the dose of 10 μM in A549 cells for 15 h and 10 μM in PC-9 cells for 20 h, the cells were harvested to extract proteins. Then, a BCA kit (Servicebio, Wuhan, China, Cat No: G2026) and SDS-PAGE (Servicebio, Cat No: G2003) were used to quantify proteins. Five nanograms of proteins were loaded per well for immunoblotting. Polyvinylidene difluoride membranes (Servicebio, Cat No: G6015-0.45) were blocked with 5% non-fat milk in TBST buffer; incubated with primary antibodies against GAPDH (1:2000, Servicebio, Cat No: GB15004), ACTIN (1:2000, Servicebio, Cat No: GB15003), p-GSK-3β (1:1000, Servicebio, Cat No: GB114582), cyclin A1 (1:500, ABCLONAL, Cat No: A14529), cyclin-dependent protein kinase 1 (1:1000, CDK1, Servicebio, Cat No: GB11398), cyclin-dependent protein kinase 2 (1:1000, CDK2, Servicebio, Cat No: GB112129), and cyclin B1 (1:1000, Servicebio, Cat No: GB112098) at 4 °C overnight; and then incubated with HRP-conjugated secondary antibody (1:5000) for 30 min at 25 °C. The signals were visualized by using an ECL kit (Servicebio, Cat No: G2014). AIWBwell^TM^ (Servicebio) was employed to calculate the intensity of the signals in each sample.

### 4.9. Luciferase Reporter Assay 

A luciferase reporter assay was used to validate the combination of miR-4516 with Wnt 8B. A total of four miRNA-4516 binding sites (131-137, 504-510, 792-798, and 799-805) in the 3′-UTR of the Wnt 8B gene were mutated. Wnt 8B-WT and Wnt 8B-MT were synthesized and cloned into pmirGLO reporter vectors upstream of the luciferase reporter gene. Then, miR-4516 mimics and NC mimics were synthesized and mixed with cloned pmirGLO reporter vectors in 50 µL of basic DMEM. 293T cells were cultured at a density of 2 × 10^4^ cells/well in 96-well culture plates and co-transfected with NC mimics + Wnt 8B-WT, miR-4516 mimics + Wnt 8B-WT, NC mimics + Wnt 8B-MT, and miR-4516 mimics + WNT 8B-MT using LipofectamineTM 2000 (Invitrogen, Carlsbad, CA, USA) for 4 h. Forty-eight hours post-transfection, the cells were lysed and firefly luciferase activity was measured using the Dual Luciferase^®^ Reporter Assay System (Promega, Madison, WI, USA) normalized to renilla luciferase as the internal reference.

### 4.10. Expressions of miR-4516 and Its Target Genes in the Wnt Signaling Pathway in Human Lung Adenocarcinoma

The TCGA and GTEx databases were used to detect the expressions of miR-4516 and Wnt 8B, FZD2, DVL3, and FOSL1 in the Wnt signaling pathway and the downstream gene expressions of CDK1, CDK2, CCNA1, and CCNB1, which have a greater association with cell cycle and apoptosis, in tumor tissue from primary LUAD and normal lung tissue. Additionally, the association between mRNA expressions of the target genes and the overall survival of patients with LUAD was analyzed.

### 4.11. Statistical Analysis

All data are shown as the mean ± the SD. SPSS 23.0 software was employed to perform the statistical analysis. Intergroup comparisons were analyzed using one-way ANOVA or *t* tests. *p* values less than 0.05 were considered statistically significant.

## Data Availability

Data are contained within the article and Supplementary Materials.

## Supplementary Materials

The following supporting information can be downloaded at https://www.mdpi.com/article/10.3390/molecules29122797/s1, Figure S1: GHaK did not exhibit cytotoxic effects on A549 and PC-9 cells at the dose of 5 μM after 24 h of treatment; Table S1: Primers of differentially expressed miRNAs targeting the Wnt signaling pathway; Table S2: Primers of target genes in the Wnt signaling pathway.
