# Supplementary material for: Temporin-GHaK Exhibits Antineoplastic Activity against Human Lung Adenocarcinoma by Inhibiting the Wnt Signaling Pathway through miRNA-4516
Source: Molecules. 2024 Jun 12;29(12):2797. doi: 10.3390/molecules29122797 (PMC11206823; doi:10.3390/molecules29122797)
Supplement: Supplementary file 1 [file molecules-29-02797-s001.zip › molecules-3052982-supplementary.pdf]

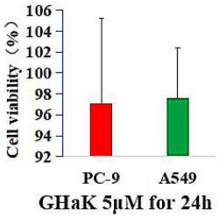

Figure S1. GHaK did not exhibit cytotoxic effects on A549 and PC-9 cells in the dose of 5 μM after 24 h of treatment.

Table S1. Primers of each differentially expressed miRNAs targeting Wnt signaling pathway

Gene symbol	Forward primer/Reverse primer
U6	F:5′GCTTCGGCAGCACATATACTAAAAT3′
	R:5′CGCTTCACGAATTTGCGTGTCAT3′
miR-1296-3p	F:5′GAGAGTGGGGCTTCGACC3′
	R:5′GTGCGTGTCGTGGAGTCG3′
miR-4463	F:5′GGGGAAAGAGACTGGGGT3′
	R:5′GTGCGTGTCGTGGAGTCG3′
miR-12136	F:5′GGGGGGGAAAAAGTCATG3'
	R:5′GTGCGTGTCGTGGAGTCG3′
miR-4516	F:5'GGGGAAGGGAGAAGGGTC3'
	R: 5'GTGCGTGTCGTGGAGTCG3′
miR-4284	F:5′GGGGAAGGGCTCACATCA3′
	R:5′GTGCGTGTCGTGGAGTCG3′
miR-204-5p	F:5′GGGTTCCCTTTGTCATCC3′
	R:5′CAGTGCGTGTCGTGGAGT3′

Table S2. Primers of each target genes in the Wnt signaling pathway

Gene symbol	Forward primer/Reverse primer
β-actin	F:5' GTGGCCGAGGACTTTGATTG3'
	R:5′ CCTGTAACAACGCATCTCATATT3′
DVL3	F:5' TGCAGCGACCCAGCTATAAG 3′
	R:5′ GCAGCTCCGATGGGTTATCA 3′
FOSL1	F:5' CACATCCAACTCCAGCAACTT3′
	R:5′ CTCTGGCACAAATGGGAAATA3′
FZD2	F:5' GCGAAGCCCTCATGAACAAG 3′
	R:5′ TCCGTCCTCGGAGTGGTTCT 3′
WNT 8B	F:5' ATCAGTTTGCCTGGGACCG 3′
	R:5′ TTGGAAATCGCCTCTCCGAA 3′
